# KDiamend: a package for detecting key drivers in a molecular ecological network of disease

**DOI:** 10.1186/s12918-018-0531-8

**Published:** 2018-04-11

**Authors:** Mengxuan Lyu, Jiaxing Chen, Yiqi Jiang, Wei Dong, Zhou Fang, Shuaicheng Li

**Affiliations:** Department of Computer Science, City University of Hong Kong, Tat Chee Avenue, Hong Kong, China

**Keywords:** Molecular ecological network, Microbiome, Key driver, Delegated phenotype, Disease

## Abstract

**Background:**

Microbial abundance profiles are applied widely to understand diseases from the aspect of microbial communities. By investigating the abundance associations of species or genes, we can construct molecular ecological networks (MENs). The MENs are often constructed by calculating the Pearson correlation coefficient (PCC) between genes. In this work, we also applied multimodal mutual information (MMI) to construct MENs. The members which drive the concerned MENs are referred to as key drivers.

**Results:**

We proposed a novel method to detect the key drivers. First, we partitioned the MEN into subnetworks. Then we identified the most pertinent subnetworks to the disease by measuring the correlation between the abundance pattern and the delegated phenotype—the variable representing the disease phenotypes. Last, for each identified subnetwork, we detected the key driver by PageRank. We developed a package named KDiamend and applied it to the gut and oral microbial data to detect key drivers for Type 2 diabetes (T2D) and Rheumatoid Arthritis (RA). We detected six T2D-relevant subnetworks and three key drivers of them are related to the carbohydrate metabolic process. In addition, we detected nine subnetworks related to RA, a disease caused by compromised immune systems. The extracted subnetworks include InterPro matches (IPRs) concerned with immunoglobulin, Sporulation, biofilm, Flaviviruses, bacteriophage, *etc.*, while the development of biofilms is regarded as one of the drivers of persistent infections.

**Conclusion:**

KDiamend is feasible to detect key drivers and offers insights to uncover the development of diseases. The package is freely available at http://www.deepomics.org/pipelines/3DCD6955FEF2E64A/.

## Background

Assessment and characterization of microbiota are prevalent in human disease studies [1–3]. When the species within the microbial community interact with each other in equilibrium, serving as co-adapted colonists and providing beneficial goods and services, disruption of such alliances may induce health issue [4]. For example, the imbalance in the community could lead to bacterial overgrowth and the development of respiratory infections [5]. In this case, network analysis, for instance, differential network analysis, which identifies biomarker candidates by detecting changes in the correlation relationships between different experimental conditions [6], provides a better understanding towards disease. Thereafter, in microbiome area, molecular ecological networks (MENs) [7] can be constructed to perform network analysis for different types of actors within the microbial community, for examples, species, taxons, or phylogenetic gene markers, and they are referred to as phylogenetic molecular ecological networks (pMENs) where phylogenetic gene markers serve as the actors [8]. Similar to co-expression networks, Deng et al. proved that the MENs are scale-free and small world [7].

In a MEN, the removal of some species could be disproportionately deleterious. These species are referred to as *keystone species*. Keystone species are topologically important molecules in the MEN. Berry et al. has studied the detection of keystone species in MENs thoroughly [9]. They applied a brute-force leave-one-out strategy to evaluate the keystoneness of a species in a given MEN, and demonstrated the impact of the keystone species on species richness. They also classified keystone species according to their topological properties using linear discriminant analysis. Deng et al. proposed a method to detect keystone species from the MENs by integrating phenotype information [10]. They identified keystone species by calculating the correlation between a phenotype variable and the abundance pattern of species clusters. Researchers also considered species connected to many others in MENs as keystone species (also referred to as hub nodes) [11].

Key drivers, which are major components that drive the disease concerned MENs, provide hints to understand the mechanisms of disease and are intensively studied with RNA data. There are various of methods to identify the key drivers in a co-expression network. One method is to incorporate the annotation of genes and pathways of diseases in order to locate the key drivers by considering enrichment of statistic of genes neighborhood [12, 13]. Another category of method distinguishes important MENs by calculating associations between gene modules with meta information like phenotype and GWAS analysis [14, 15], and then detects the key drivers by measuring the genes topology effect. For example, MEGENA [16] did multiscale hub analysis and Zhang et al. examined the number of N-hob downstream nodes [17]. Those methods on detecting key drivers in RNA data analysis can be adopted to detect key drivers in MENs. Even though Portune et al. locates important microbial species and genes with the assistance of gene annotation to study the MENs [18], the annotation for microbial genes and species yet demands intensive efforts and the pathways of diseases are incomplete.

The distinction between keystone species and key drivers is that the keystone species are only topologically important, while key drivers motivate disease associated networks. MENs of diseases can be different compared to those from healthy individuals. By analyzing the factors driving the differences, we can uncover the development of the disease.

Inspired by key drivers analysis with RNA data and keystone species studies in MENs, we proposed a method to perform key drivers analysis without the availability of annotation information. Given a microbial abundance profile, we first construct the MEN, in which the nodes represent the microbial species or phylogenetic gene markers and the edges capture the associations between their respective nodes. Then we divide the MEN into multiple subnetworks and extract the subnetworks that are most relevant to the disease by calculating the associations between subnetworks and phenotype variables. A single phenotype variable could be insufficient to capture the changes in disease networks from healthy networks and it can be biased. To address this issue, we applied principal component analysis to extract delegated phenotype, which is more robust. Last, our method detects the key driver based on PageRank, which utilizes node topological properties within each extracted subnetwork. It captures the global link structure of subnetworks thus outperforms statistical algorithms that only use local information.

There are multiple ways to calculate inference of MENs, of which two of the most popular ways are Pearson correlation coefficient (PCC) and mutual information (MI) [19]. A review of correlation detection strategies in MENs [20] suggests that although some methods outperform others, the inference calculating method still needs further improvement. To reduce the effect of the high proportion of zero counts, Paulson et al. applied a mixture model that implemented a zero-inflated Gaussian (ZIG) distribution of mean group abundance for each taxonomic feature to do differential abundance analysis. Experiments show the improvement of mixture model compared to other models, for instance, DESeq, edgeR and Kruskal-Wallis test [21, 22]. Inspired by the above trials of solving rare microbes issues with mixture models, we proposed to construct the network by multimodal mutual information (MMI) [23] under the assumption of the Gaussian mixture model. In KDiamend, we implemented both PCC and MMI to infer the associations between nodes in the MENs. However, correlation-based methods, like PCC, have their limitations. To be more specific, it is hard to distinguish correlation with causation [24]. There are many other arbitrary methods to construct networks, like Bayesian network [24] and WGCNA [25], which apply topology overlaps to measure the similarities between nodes. These various methods can be implemented to construct the network as potential options in our framework. Nevertheless, it is out of the range of this work.

Our main contribution is that we refined the framework of key driver detection, and proposed delegated phenotype to capture the changes in disease networks from healthy networks. To validate our method, we performed experiment based on simulated data. Then, we tested KDiamend with two real microbiome datasets. We conducted key drivers analysis on Type 2 diabetes (T2D) and Rheumatoid Arthritis (RA), whose data are from gut microbiome and oral microbiome respectively. For each disease, we also compared experiment using PCC and MMI as two different inference methods, and acquired both consensus and divergence. Experiments of the two inference methods identified multiple identical phylogenetic gene markers and identified consensus pattern of disease-associated networks, indicating the robustness of our framework. On the other hand, the two different inference methods also led to specific findings, providing us with various aspects to study the mechanisms of diseases. We detected six T2D-relevant subnetworks and identified key drivers for each of them correspondingly. The identified key drivers include IPR006047, IPR018485 and IPR003385 related to the carbohydrate metabolic process, while the carbohydrate metabolic process is an important issue during the development of T2D [26]. In addition, we also detected key drivers for RA. Both PCC and MMI experiments located multiple InterPro matches (IPRs) which are related membrane and infection. Six subnetworks were extracted by PCC, containing IPRs concerned with immunoglobulin, Sporulation. Three subnetworks were detected by MMI, with IPRs about biofilm, Flaviviruses, bacteriophage, etc. The result is inspiring since the development of biofilms is regarded as one of the drivers of persistent infections [27] and some biofilms-growing bacterias contribute to RA [28].

## Methods

Our method is to detect the key drivers which drive the diseases related networks in the microbial community. The key drivers can be microbial species or phylogenetic gene markers. For simplicity, we present our method with nodes as genes in the subsequent descriptions.

**Fig. 1:**
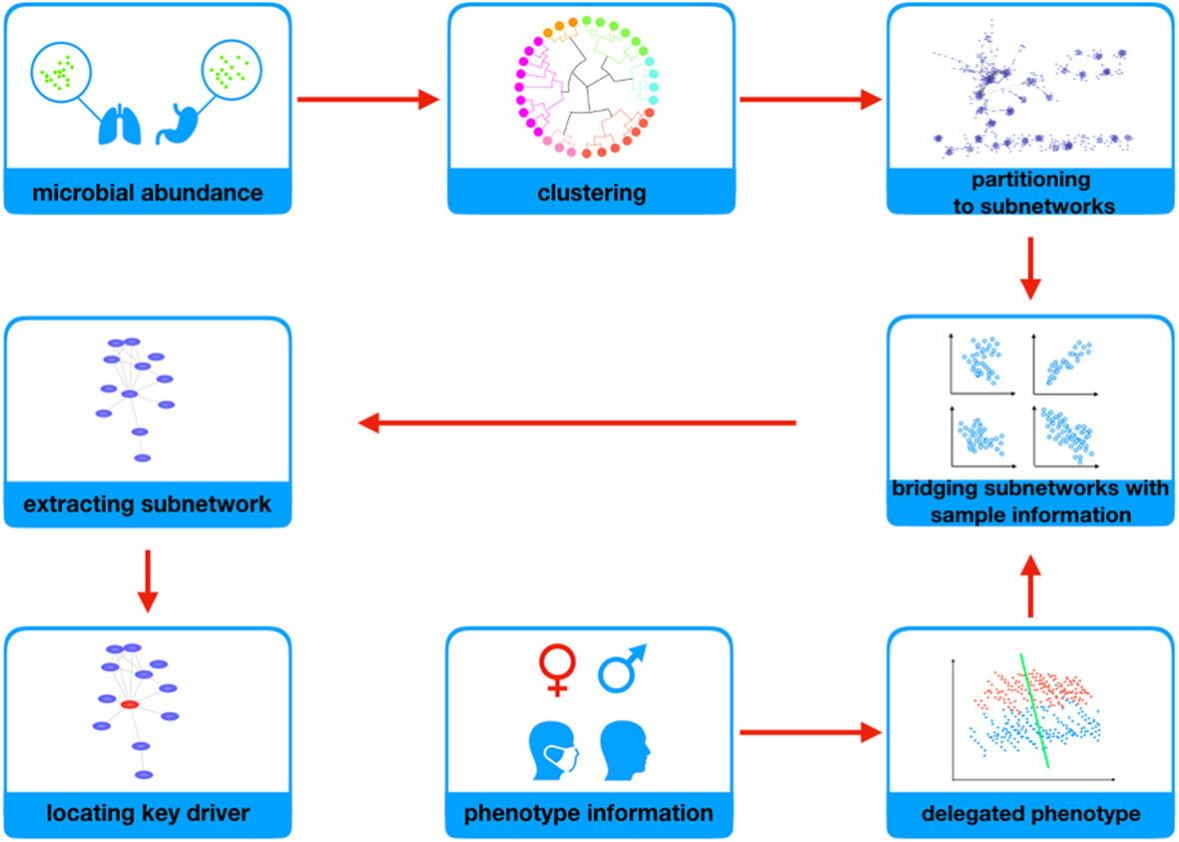
Flowchart. First, we build a MEN and cluster genes into multiple subnetworks. After that, we summarize the phenotype variables and connect it to subnetworks. Then, we locate key drivers through PageRank

The detection of key drivers consists of following steps (see Fig. 1). First, we construct a MEN to represent the relationship between genes based on microbial abundance profiles and infer the weight of each edge. Second, we cluster the genes and partition the MEN into multiple subnetworks. Third, we analyze the phenotype variables and extract the delegated phenotype. By computing the associations between subnetworks and delegated phenotype, we obtain subnetworks that are most related to the disease. Last, based on PageRank, we identify actors with top influence over others in each subnetwork as key drivers.

### Inference method

In KDiamend, we provide two ways to compute distances between genes. The first one is PCC, which is the most popular way to capture similarities between genes. In addition, inspired from the inference of gene regulatory network in RNA analysis and mixture models in the microbiome analysis, we adopted the MMI and normalization processes in Context Likelihood of Relatedness(CLR) [29]. MI, which uses the mutual dependency and common uncertainty as for the measurement of connection between genes, does not assume linear, or continuous dependence like correlation [19, 30], so it can detect interactions which might be missed by PCC. MMI, under the assumption of the Gaussian mixture model, is for dealing with the high proportion of zero counts issue. The adopted CLR, which considers the context of the whole network and eliminates noises from the background, makes MMI more tolerant for noises when measuring the interactions.

At the beginning, we have an abundance matrix *E*, which contains abundance value of n genes in m samples. For each gene *i*, we have a vector of *X*_*i*_=(*E*_*i*1_,*E*_*i*2_,…,*E*_*im*_). The strength of the relationship between gene *i* and gene *j* can be measured by PCC:

1 $$ PCC(i,j)=\frac{cov(X_{i},X_{j})}{\sigma_{i},\sigma_{j} },  $$

where *cov*(*X*_*i*_,*X*_*j*_)is the covariance of *X*_*i*_ and *X*_*j*_, and *σ*_*i*_ is the standard deviation of *X*_*i*_. The adjacency matrix A of network can be generated from *A*_*ij*_=*PCC*(*i*,*j*). Then the distance between gene *i* and gene *j* can be interpreted as *D*_*ij*_=1−|*PCC*(*i*,*j*)|.

Apart from PCC, we also implemented MMI [23]. First, we decomposed *X*_*i*_ into *c*_*i*_ bins as *X*_*i*,1_, …, $X_{i, c_{i}}$. In this case, *X*_*i*_ was distributed according to the following function:

2 $$ f_{X_{i}}(x)=\sum_{k=1}^{c_{i}}\pi_{i, k}g_{X_{i, k}}(x),  $$

where $g_{X_{i, k}}(x)$ (1≤*k*≤*c*_*i*_) denotes the density function for *C*_*i*,*k*_, and *π*_*i*,*k*_ is the proportion for each sample in *C*_*i*,*k*_. As proved in former work [23], assuming that *X*_*i*,*k*_ fits in a Gaussian distribution, we can estimate the mutual information between *X*_*i*_ and *X*_*j*_ as:

3 $$ MMI(X_{i},X_{j})=MMI^{O}(X_{i},X_{j})+MMI^{I}(X_{i},X_{j}).  $$

The “outer” MI, *MMI*^*O*^(*X*_*i*_,*X*_*j*_), captures discretized dependency, while the “inner” MI, *MMI*^*I*^(*X*_*i*_,*X*_*j*_), refers to the weighted aggregation of MI for each bin.

After computing MMI between all the genes and get a matrix *M*, we normalized the distance between gene *i* and gene *j* by: $CLR(Z_{i},Z_{j}) = \sqrt {\left (Z_{i}^{2},Z_{j}^{2}\right)}$ where *Z*_*i*_ and *Z*_*j*_ are z-scores of *M*_*ij*_ taking *M*_*i*_ and *M*_*j*_ as background respectively [29]. Then we applied hierarchical clustering and partitioned the MEN into multiple subnetworks according to the distance between genes.

### Delegated phenotype

To best capture phenotype change in disease networks from healthy networks, we generated delegated phenotype by rotating Coordinates in the PC space of phenotype variable matrix *S*. Each row in *S* represents a sample while each column refers to a phenotype variable, such as gender, age, disease state, etc. If the properties are nonnumerical, we converted data into numbers before further analysis. Suppose one column *v* in *S* indicates whether each sample is collected from a person with or without this disease. That is *v*=(*v*_1_,*v*_2_,…,*v*_*k*_,…,*v*_*m*_),*v*_*k*_∈(*Y*,*N*),1<*k*<*m* where *m* is the number of samples, Y indicates that this sample is collected from a person with the disease, and N means not.

We applied principal component analysis (PCA) to conclude *S*. We consider the first two principal components (PCs) to be enough for explaining disease variability, since the number of phenotype variables is relatively small in our test data. When phenotype data are more complicated, we may need extra analysis to decide the number of PCs we use to conclude delegated phenotype. We investigated the first two PCs and plotted samples in the coordinate of PC1 and PC2, regarding every sample as a point. Consequently, we got m points and each point refers to one sample. The coordinates for point k, is expressed as (*x*_*k*_,*y*_*k*_). We rotated *PC* to *PC*^′^ and make sure it has the largest correlation with *v* upon rotation. *PC*^′^ can best explain variability related to the disease. The angle of rotation is the *θ* that maximize *f*(*θ*).

4 $$\begin{array}{*{20}l} {}f(\theta)\! =\!\! \sum_{k, v_{k} \in N}(x_{k} cos \theta_{k} + y_{i} sin \theta_{k}) \,-\, \sum_{k, v_{k} \in Y}(x_{k} cos \theta_{k} + y_{k} sin \theta_{k}) \end{array} $$

That is to say, the angle between *PC*1 and *PC*1^′^ is:

5 $$\begin{array}{*{20}l} \theta=arctan \frac{\sum_{k, v_{k} \in N}x_{k}-\sum_{k, v_{k} \in Y} x_{k}}{\sum_{k, v_{k} \in N}y_{k}-\sum_{k, v_{k} \in Y} y_{k}} \end{array} $$

For example (see delegated phenotype in Fig. 1), blue points represent *v*_*k*_=*Y* and red points represent *v*_*k*_=*N*. By calculating *θ*, we got the line which implies the direction most correlated to the disease state.

We acquired delegated phenotype *PC*^′^, which has the largest correlation with the disease state and outperforms other single variables on explaining the variability of phenotype information at the meantime. For every subnetwork, we concluded the abundance pattern of genes as eigengenes [31]. Then we bridged the subnetworks to phenotype information by calculating the correlation between eigengenes and *PC*^′^. Subnetworks which have strong relationships to *PC*^′^ are extracted as disease-relevant subnetworks.

### Identifying Key driver

Last, in every extracted disease relevant subnetwork, we applied network topology analysis and assigned every gene a PageRank score. PageRank (PR) ranks the nodes in a graph according to the structures of links with others and is used by Google’s search engine to compute rankings of websites. In this algorithm, the score for one node can be affected by its neighbors [32] and if one’s neighbors have high scores, its score increases iteratively [33].

As stated in [34], letting *F*_*u*_ be the nodes linking to the node, *B*_*u*_ be the nodes linked from it, and *N*_*u*_=|*F*_*u*_| be the magnitude of *F*_*u*_. Besides, considering there might be other factors towards the ranking, let *E*(*u*) be the vector concerned with some of the rank. Then, the PageRank of the node is defined as.

6 $$ R(u)=c\sum_{\upsilon \in B_{u}} \frac{R(\upsilon)}{N_{\upsilon} }+cE(u)  $$

## Results

### Gut microbiome

We tested our method with real microbiome datasets and compared PCC with MMI in this framework. First, In order to detect key drivers for T2D, we downloaded processed InterPro matches (IPR) abundance data from EBI (SRP008047), which is gut metagenome (microbiome) data from Chinese samples. InterPro [35] provides a functional analysis of protein sequences by classifying them into families and predicting the presence of domains and important sites. The phenotype information of the dataset is provided in related paper [1]. We used the 145 samples from stage one.

**Fig. 2:**
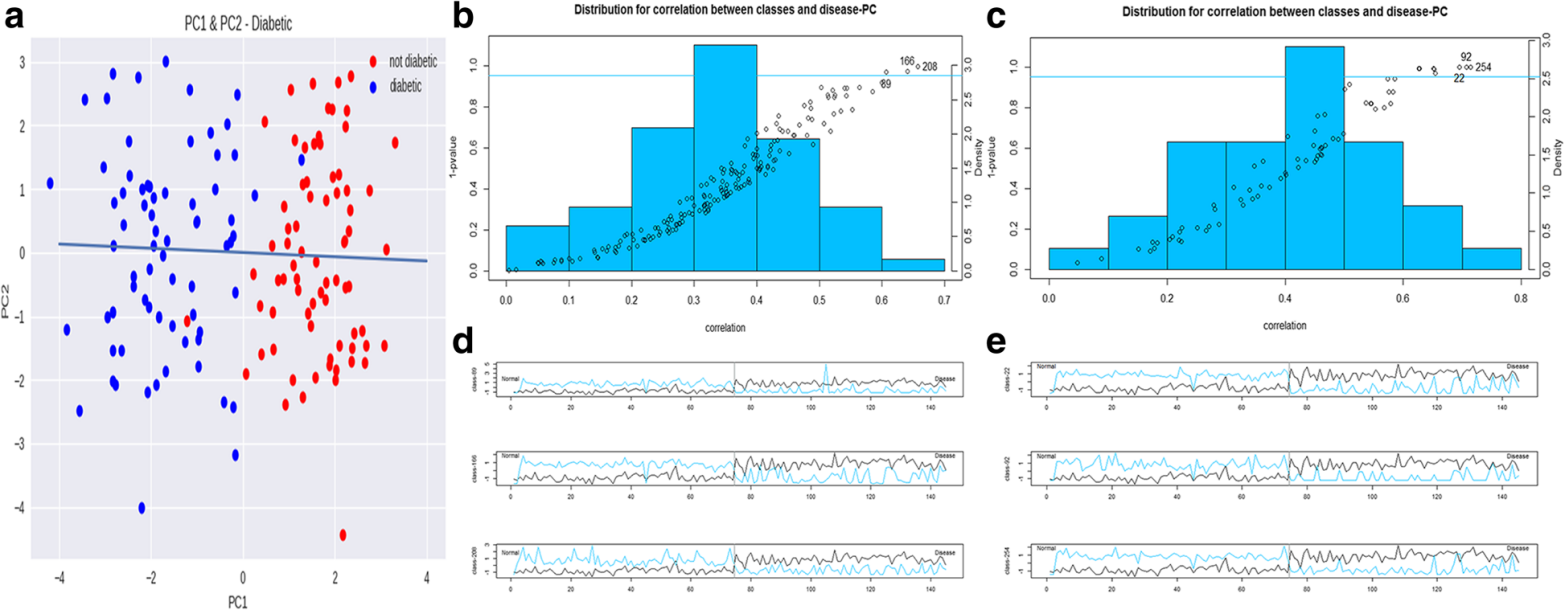
Experiment results for type 2 diabetic. **a** The delegated phenotype. **b**, **c** Distribution for correlation between subnetworks and delegated phenotype. Every point refers to a subnetwork. The x-axis of the point refers to the correlation between this subnetwork and the delegated phenotype. The y-axis of the point refers to the 1 −*p*-value for the correlation. The y-axis on the right is for the histogram of these correlation. **b** is for PCC experiment and **c** is for MMI experiment. **d**, **e** Plots for eigengene and delegated phenotype. Blue line refers to the z-score of eigengene for that subnetwork. X-axis refers to samples. **d** is for PCC experiment and E is for MMI experiment

We first trimmed IPRs with low abundance in relative abundance matrix and then applied quantile normalization [36, 37]. By computing PCC and MMI between pairs of genes, we reconstructed a MEN and conducted clustering to partition the MEN into multiple subnetworks. For all subnetworks, eigengenes were calculated by selecting the first PC of abundance. The eigengene was used for summarizing the abundance pattern in each subnetwork and to bridge it with phenotype information. On the other hand, we digitalized the phenotype matrix and applied PCA to it. We generated delegated phenotype by rotating coordinates in the PC space to best capture the phenotype change in disease networks from healthy networks. We extracted three subnetworks for PCC experiment and three subnetworks for MMI according to the correlation between eigengenes and delegated phenotype and the corresponding *p*-value (see Fig. 2). The *p*-value for correlation of each subnetwork was calculated by permuting the same number of genes from the dataset and calculating the correlation between the permuted eigengene and the delegated phenotype. After repeating 1000 times, the rank of the real correlation for the subnetwork is regarded as *p*-value.

**Fig. 3:**
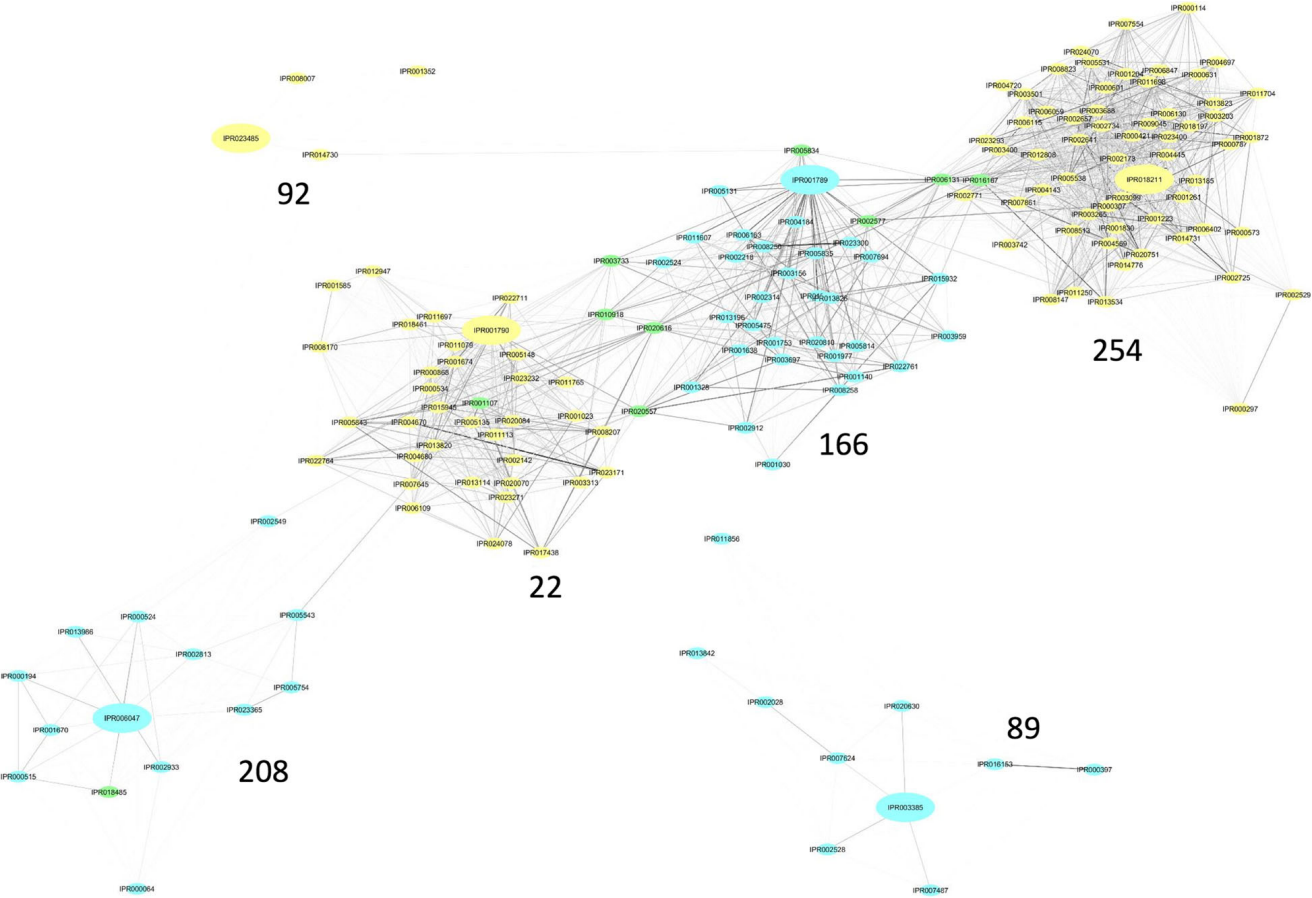
Extracted networks for T2D. Detected T2D-relevant subnetworks for PCC and MMI experiment. Blue and yellow nodes refer to IPRs identified by PCC and MMI, while green ones refer to IPRs identified by both methods. The weight calculated by PCC and MMI was normalized to the same scale. The corresponding subnetworks are labeled. Nodes with top PageRank in each subnetwork are enlarged

PCC experiment and MMI experiment detected 11 consensus IPRs which scattered in three subnetworks for MMI and two subnetworks for PCC. Consequently, the interaction generated from two types of inference connects these five extracted subnetworks together and merges them into one large community (see Fig. 3). Most of the consensus IPRs in the merged community are associated with the metabolic process and the catalytic activity which implies that the process is relevant to the disease. More specifically, two experiments both identified IPR018485 which participates in the carbohydrate metabolic process with the phosphotransferase activity and is active in carrying out ATP-dependent phosphorylation [38]. The extracted disease-relevant subnetworks in T2D are about the carbohydrate metabolic process and phosphorelay.

In addition, for PCC experiment, key drivers in subnetwork 89 and subnetwork 208 are related to the carbohydrate metabolic process, including IPR006047, IPR018485, and IPR003385. The key driver in subnetwork 166 is IPR001789, which plays a role in phosphorelay signal transduction system. MMI also detected IPR018211, IPR005538, IPR003501, and IPR001790 which are related to phosphorelay. PCC and MMI both identified IPRs related to the carbohydrate metabolic process and phosphorelay.

### Oral microbiome

We applied our method to oral microbiome to detect the key drivers in microbial community related to dysbiosis in Rheumatoid Arthritis (RA). The abundance data was downloaded from EBI (ERP006678). Information of phenotype variables for different individuals was acquired from published paper [2]. We mapped the samples downloaded from EBI with the individual ID and got 49 oral microbial samples in total. Among them, 27 samples were collected from patients with RA in different disease states, 22 samples, used as the control, were collected from people without RA. 21 of them are saliva samples and 28 are dental samples.

We first conducted filtering and then applied normalization to avoid noises. After that, we constructed the MEN by computing similarities between all pairs of IPRs. Then we partition the MEN into multiple subnetworks by clustering.

Similar to the analysis for T2D, we processed the phenotype matrix and detected subnetworks most related to RA. First, we removed phenotype variables with more than 1/3 missing values. Then, for remaining phenotype variables, we conducted imputation using R package MICE [39]. By computing correlation between delegated phenotype and eigengenes of subnetworks, we extracted six subnetworks most related to RA using PCC and three subnetworks using MMI. Finally, we identified key drivers for detected disease associated subnetworks correspondingly.

**Fig. 4:**
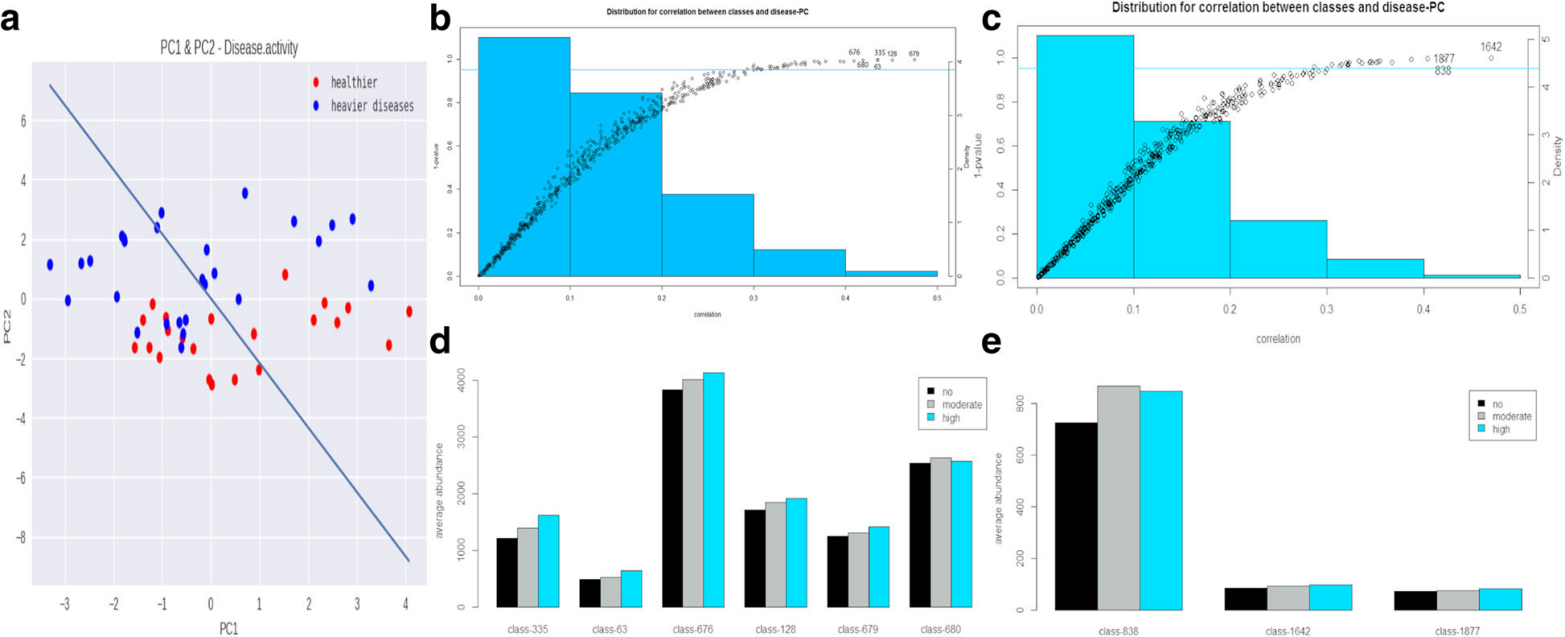
Experiment results for Oral experiment. **a** The delegated phenotype. **b**, **c** Distribution for correlation between subnetworks and delegated phenotype. Every point refers to a subnetwork. The x-axis of the point refers to the correlation between this subnetwork and the delegated phenotype. The y-axis of the point refers to the 1 −*p*-value for the correlation. The y-axis on the right is for the histogram of these correlation. **b** is for PCC experiment and **c** is for MMI experiment. **d**, **e** Bar plot for the average abundance of extracted subnetworks. The relative abundance is different in different disease states

We applied key drivers analysis for RA using PCC and MMI as two different inference methods respectively. Both experiments show IPRs, in extracted associated subnetworks, have higher abundance in disease state than in normal state (see Fig. 4). For PCC experiment, annotation shows that most IPRs in subnetwork 335 and 63 are about cell membrane while most IPRs in subnetwork 676, 128, 679 and 680 are about replication and cell growth. Functions for IPRs were inferred according to keywords and Gene Ontology (GO) mentioned in InterPro [35]. Moreover, subnetwork 335 also contains IPR014879(Sporulation initiation factor Spo0A, C-terminal) and IPR013783 (Immunoglobulin-like fold). IPR013783 is about immunoglobulin molecules and T-cell receptor antigen [40, 41], while RA is a disease caused by compromised immune systems [42].

For MMI experiment, subnetwork 1642, which has the largest correlation with delegated phenotype, contains multiple IPRs about biofilm: IPR024487, IPR019669, and IPR010344. There are totally 24 IPRs in this subnetwork and top 5 of them are IPR003496, IPR024205, IPR008542, IPR010344, and IPR019669. Specifically, IPR010344 plays a role in biofilm formation and IPR019669 participates in single-species biofilm formation on the inanimate substrate. The development of biofilms is one of the drivers of persistent infections [27]. Some bacteria, when growing in the biofilm, e.g., Porphyromonas gingivalis in dental plaque, can become destructive and may contribute to RA [28]. Besides, subnetwork 1642 also contains IPR013756, associated with Flaviviruses, and IPR009774, related to hypothetical Streptococcus thermophilus bacteriophage, which hints the infection process in this subnetwork.

## Discussion

### Noise tolerance of delegated phenotype

**Fig. 5:**
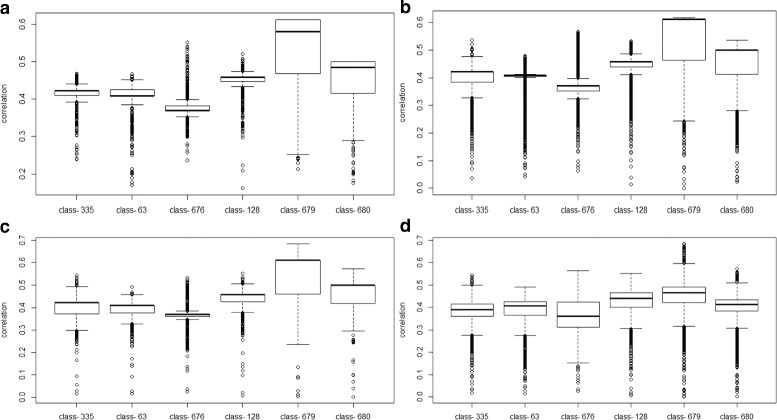
Noise tolerance of delegated phenotype. **a** The first box plot refers to combination with removal of one, two, or three phenotype variables. **b** The second refers to the removal of six phenotype variables. **c** The third refers to the removal of ten phenotype variables. **d** The last one refers to the overall performance with all these combinations

To test whether our delegated phenotype is robust when phenotypes are deficient, we tried every combination of phenotypes with removing 1,2,3,6,10 of them from the phenotype variables matrix of RA, and generated delegated phenotype for each of them. Then we calculated the correlations between those generated delegated phenotypes and extracted subnetworks. The result is promising and these extracted subnetworks have high correlation values in most cases (see Fig. 5).

### Performance of PageRank in searching the key driver

**Fig. 6:**
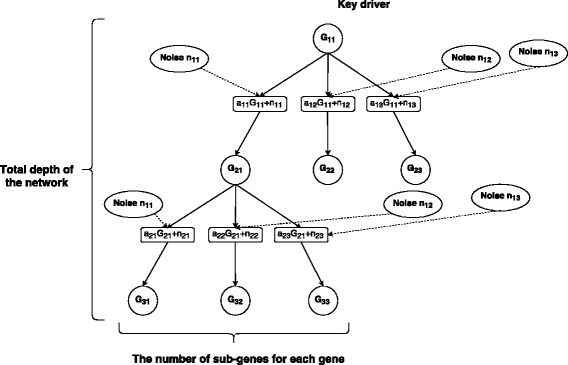
The structure of simulated network. The simplified network can be transferred into a tree

We tested the performance of PageRank on a simulated dataset. At the beginning, we named the driven relationship as sub-gene relationship. We simplified the network by assuming that one gene could only be driven by one gene. Linear function is used to represent the driven relationship. i.e. *y*=*Ax*+*n*, where *x* and *y* denotes expression levels for gene and its sub-gene and *n* is the noise following the normal distribution with 0 mean. There are three parameters for the simulation algorithm: the number of sub-genes for each gene, the depth of the network and the noise level. Here, we used the variance to define the noise level and the variance of noise is *βx*. The structure of the simulated network and three parameters are shown in Fig. 6.

**Fig. 7:**
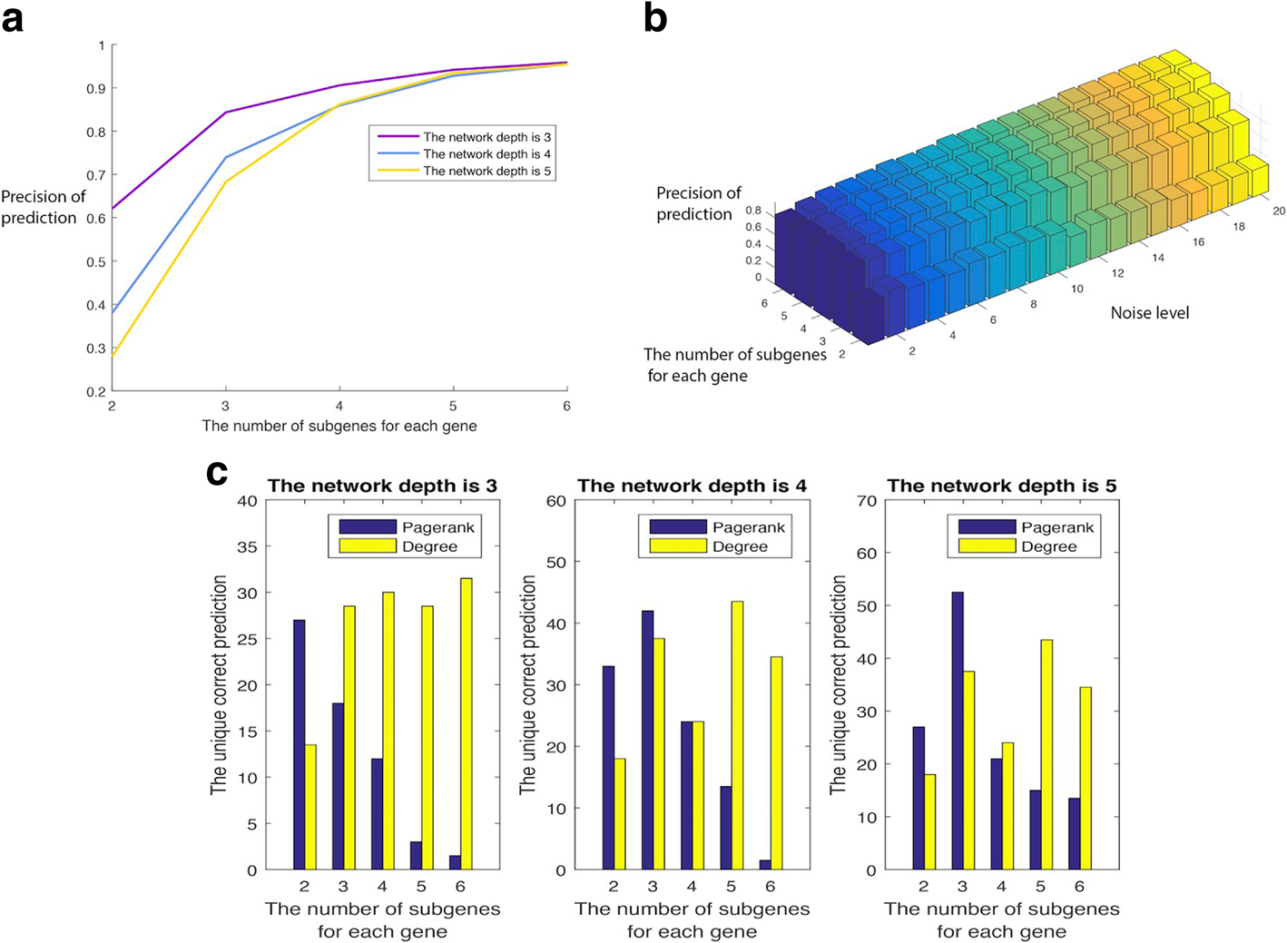
Testing results for PageRank algorithm to find the key driver. The first two figures show the prediction precision of PageRank under different simulation parameters. **a** is related to the network depth and the number of sub-genes. **b** is for the noise level and the number of sub-genes. The third figure compares the performances of PageRank and degree algorithm. We counted cases where only one algorithm found the key driver correctly

We generated the simulated data with various parameters. For each parameter group, 100 samples were produced. We compared the performance of PageRank with the degree algorithm that locates the key driver with the highest degree. As shown in Fig. 7, the noise level has little effect on prediction precision. The result of the degree algorithm also follows this pattern. To compare these two algorithms, we collected the cases where only one algorithm correctly found the key driver and the result is shown in *C*. Since when the number of sub-genes is large, both algorithms have high prediction precision, we focus more on cases where the sub-gene number is relatively small. In this situation, the PageRank has better performance.

### Application to Alzheimer’s disease

To further validate our method is capable of detecting key drivers of disease, we applied KDiamend to Alzheimer’s Disease (AD) with analyzing RNA expression profiles, which were downloaded from GEO(GSE44770) [17]. Both of PCC and MMI experiments identified FBXL16 and OLFM1. FBXL16 related pathways are Innate Immune System and Class I MHC mediated antigen processing and presentation, while researches have shown that the activation of the Innate Immune System plays a crucial role in promoting AD [43]. OLFM1 is related to nervous system development and Neuroblastoma [44]. Besides, PCC experiment also identified RPS4Y1 and PITPNB as key drivers for extracted disease associated subnetworks. MMI experiment also identified KAZALD1, OR4A47, RNASE11, TXNDC2, 7-Mar, RTN4, TSPAN9, PCNP and PPP2R2C. More specifically, RTN4 is related to Demyelinating Disease [45] and KAZALD1 is related to Lobar Holoprosencephaly [46]. These experiments show a possible application of our method. It is capable of detecting key drivers in the network inferred from not only the microbial abundance profile but also other kinds of abundance data, like RNA expression or proteomics.

## Conclusion

We proposed a novel method to detect key actors who drive the disease concerned MENs, which helps to understand microbial factors relevant to the certain disease. We divided the MENs into multiple subnetworks and then, instead of detecting important genes based on pathways or gene annotations, we extracted subnetworks which are most relevant to disease by utilizing the correlation between the patterns of abundance profiles and the delegated phenotype. Lastly, we identified key drivers based on PageRank.

We tested our method with two real microbial datasets. We detected that the disease-relevant subnetworks in T2D are related to the carbohydrate metabolic process and phosphorelay, while RA-relevant subnetworks are related to membrane, cell growth, and infection. The extracted subnetworks for RA include IPRs concerned with immunoglobulin, Sporulation, biofilm, Flaviviruses, bacteriophage, etc. Then we located corresponding key drivers for extracted disease-relevant subnetworks. Besides microbial data, we also tested our method with gene expression profiles to identify key drivers for AD and the outcome was inspiring. Experiments show our method is capable of detecting key drivers and providing hints to understand the mechanisms of diseases.
